# Ecological and Demographic Influences on the Prevalence of Sleep Disorders and Their Impact on Cognitive Decline

**DOI:** 10.7759/cureus.80162

**Published:** 2025-03-06

**Authors:** Muddsar Hameed, Zainab Salahuddin, Ismat Ullah Abid, Haniya Ihsan, Umair Nasir, Aleena Safdar Bukhari, Abdulrahman AlQaderi, Mehroz Zameer Khan, Dalia Emara, Budoor Bin Bahar, Abdullah Salem Alsharif, Sumayyah Leila Zaman, Alyazah Alsuwaidi

**Affiliations:** 1 Department of Neuroscience, Brain Tech Clinic and Research Center, Islamabad, PAK; 2 Department of Psychology, Shaheed Zulfikar Ali Bhutto Institute of Science and Technology University, Islamabad, PAK; 3 Department of Public Health, Health Services Academy, Islamabad, PAK; 4 Department of Medicine, CMH Lahore Medical College, Lahore, PAK; 5 Department of Medicine, Glenfield Hospital, Leicester, GBR; 6 Department of Medicine, Federal Medical and Dental College, Islamabad, PAK; 7 Department of Medicine, RAK Medical and Health Sciences University, Ras Al-Khaimah, ARE; 8 Department of Medicine, Islamabad Medical and Surgical Hospital, Islamabad, PAK; 9 Department Orthodontics, Dubai Health, Dubai, ARE; 10 Department of Medicine, University of Sharjah, Sharjah, ARE

**Keywords:** air pollution, cognitive decline, ecological factors, environmental noise, sleep disorders, sleep quality

## Abstract

Introduction: Sleep disorders are increasingly recognized as significant public health concerns, influencing cognitive function and overall well-being. While previous studies have established a connection between sleep disturbances and cognitive decline, little is known about the impact of ecological factors such as noise pollution, air quality, and temperature on sleep health. This study examines the relationship between ecological influences, the prevalence of sleep disorders, and their impact on cognitive functioning in various demographic groups.

Methods: This cross-sectional study included 150 participants aged 18 and older. Data were collected using self-reported ecological and sleep quality questionnaires and the Mini-Mental State Examination (MMSE) to assess cognitive function. Statistical analysis was conducted using IBM SPSS Statistics for Windows, Version 26 (Released 2019; IBM Corp., Armonk, New York, United States), with chi-square tests (χ^2^), independent sample t-tests, ANOVA, and correlation analysis to assess the relationship between ecological factors, sleep disorders, and cognitive outcomes.

Results: Gender differences were observed, with males showing lower rates of moderate cognitive impairment (n = 12) compared to females (n = 30), although sleep quality differences were not statistically significant (t(148) = 0.51, p = 0.61). Older adults exhibited significantly lower MMSE scores (χ^2^ = 135.0, p = 0.00), indicating more significant cognitive impairment than middle-aged adults. Ecological factors such as high environmental noise and poor air quality were negatively correlated with sleep quality (r = -0.79, p < 0.05) and cognitive function (r = -0.90, p < 0.01).

Conclusion: This study highlights the substantial impact of ecological and demographic factors on sleep health and cognitive function. Poor sleep quality, influenced by noise pollution, air quality, and temperature fluctuations, is associated with cognitive decline, particularly in older adults. Maintaining an optimal sleep duration of approximately seven hours may serve as a protective factor against cognitive impairment. Given the observed associations, targeted interventions such as noise reduction policies, improved air quality measures, and urban planning for better sleep environments could help mitigate sleep-related cognitive decline. Future longitudinal studies incorporating objective sleep monitoring techniques are recommended to strengthen causal inferences.

## Introduction

Sleep disorders are increasingly recognized as a significant public health concern, with far-reaching implications for cognitive functioning and overall well-being. Quality sleep is crucial for maintaining good health [1]. Sleep disorders are frequent and can seriously affect the general health and condition of the patients [2]. The COVID-19 pandemic has affected the quality of sleep; problems such as insomnia, sleep fragmentation, and increased levels of anxiety and depression, especially in women, those with pre-existing psychiatric conditions, and individuals with limited social support, have resulted from this [3].

Sleep disorders in psychiatric patients are relatively pervasive. The prevalence of many disorders, like narcolepsy, sleep-breathing disorders, restless leg syndrome, and others, was estimated to be 40.75%. Symptoms like these were significantly associated with age, low physical activity, and anxiety disorders [4]. Dietary nutrition is supposed to play an important role in sleep wellness; nutritional components were even found to promote sleep. However, the link between specific foods and sleep is complicated due to individual differences in metabolic capabilities [5].

Individual sleep is shaped by both social and societal factors [6]. Sleep disorders like insomnia and excessive daytime sleepiness (EDS) have a high prevalence and are underdiagnosed, which causes more significant impairment in cognitive function, social life, and working environment [7].

Environmental factors such as family dynamics, neighborhood safety, and pollution affect sleep patterns and lead to sleep disorders [8]. Environmental noise from transportation is the leading cause of sleep disturbance. These disturbances have observable biological effects, including stress responses and alterations in sleep architecture. Poor quality sleep due to noise exposure has been associated with daytime sleepiness, mood changes, and impairments in cognition. Long-term exposure contributes to severe cardiometabolic and psychiatric diseases [9].

Research highlights that the risk of sleep disturbance was higher with greater aircraft noise levels and was significantly more adverse at higher exposures [10]. Significant correlations exist between work-related stressors like job control, noise, heat stress, and respirable dust with sleep disturbances. This includes noise, which has been related to obstructive sleep apnea [11]. Higher air temperature and CO₂ concentration harmed slow-wave sleep, while noise levels decreased sleep efficiency [12].

Studies show that sleep quality was best at 32°C, with poor sleep at higher temperatures mainly affecting sleep duration and shallow sleep. Subjective measures indicated that temperature influences calmness, sleep satisfaction, and sleep onset [13]. An association exists between poor indoor environmental quality (IEQ) and cognitive decline, though its significance depends on specific factors and cognitive functions [14].

Consistent patterns associate chronic air pollution exposure with diminished cognitive functions, notably visuospatial ability, and risk for dementia [15]. People who slept fewer than four hours or more than 10 hours a night experienced cognitive decline more quickly than people sleeping seven hours [16]. Sleep disturbances, such as waking after sleep onset, are associated with worse cognitive function and higher decline rates over five years. Self-reporting showed minimal association with cognition [17].

A correlation was found between rapid eye movement (REM) sleep behavior disorder (RBD) and impaired domains of cognition, which included memory and executive functions. EDS has also been reported to be associated with cognitive deficits [18]. Poor sleep quality was associated with an increased risk of cognitive impairment [19]. Two or more complaints of cognitive impairments, which are memory, naming, and calculations unrelated to objective performance, are related to sleep disturbances [20].

Understanding the interaction between ecological factors and the prevalence of sleep disorders is important to develop intervention strategies to mitigate their impact on cognitive health. The present study aims to investigate the association between ecological factors, sleep disorders, and cognitive decline.

Rationale

Sleep problems are one of the common public health problems affecting all areas of people's lives, including cognition, emotional state, and even physical well-being. Numerous studies have shown that sleep is an essential part of cognitive processes and that disturbances are associated with forgetting things, loss of attention, and, in some instances, deterioration of cognition, especially in the elderly. On the other hand, little is known about the ecological factors, including environmental conditions, socioeconomic status, and lifestyle behaviors, and how these factors impact the occurrence and growth of sleeping disorders. Ecological factors refer to external environmental conditions that influence human health and well-being, including sleep quality. These factors can be broadly categorized into physical and social factors. Physical ecological factors include noise pollution, air quality, temperature regulation, and the presence of greenery, all of which can directly impact sleep by affecting comfort and circadian rhythms. Social-ecological factors encompass neighborhood conditions, socioeconomic status, and access to healthcare, which influence stress levels and overall sleep hygiene. Together, these elements shape sleep patterns and may contribute to sleep disturbances, particularly in vulnerable populations.

Some ecological factors can also determine the quality of sleep, including noise pollution, air quality, temperature regulation, and even how much greenery there is in an area. Individuals living in different environments (urban vs. rural, high vs. low socioeconomic status) experience varying levels of these stressors, which may lead to or exacerbate sleep disorders. Furthermore, chronic sleep disturbances are known to worsen cognitive functioning, which has deleterious repercussions in the long term, especially among the older population. The study incorporates key demographic variables, including gender, education level, age group, and marital status, to analyze their potential influence on sleep patterns and cognitive impairment. The sample includes males and females, with educational attainment ranging from high school to bachelor's and master's degrees. Age distribution consists of middle adults and older adults, highlighting potential age-related differences in sleep and cognitive outcomes. Marital status is also considered, with participants categorized as single or married. These demographic factors will be utilized to explore their associations with sleep quality and cognitive impairment, providing a comprehensive understanding of the contributing variables.

This research explores how ecological influences impact sleep disorders and their subsequent effect on cognitive function to identify modifiable environmental factors that may reduce sleep-related cognitive decline.

Objectives

This study aims to investigate the relationship between sleep disorders and cognitive functioning, focusing on how poor sleep impacts cognitive function/decline, and to evaluate how ecological factors contribute to disturbed and poor sleep using self-report measures.

## Materials and methods

Study design and setting

This cross-sectional, quantitative study design explores the ecological factors that determine the onset of sleep disorders and their association with cognitive decline. The research population included only adults aged 18 years and older who consented to be part of the study. Patients who had any level of cognitive impairment, dementia, night-shift workers, pregnant females, and sleep disorder patients who were receiving treatments were excluded. The data was collected from hospitals in Rawalpindi and Islamabad between August 2024 and October 2024.

Sample size and technique

WHO sample size calculation was used with a confidence interval (CI) of 95%, an anticipated population proportion of 0.90, and an absolute precision of 0.05; our calculated sample size was 150. A non-probability purposive sampling technique was used to collect data from 150 individuals.

Data collection tools and procedure

We used different questionnaires to assess ecological influences on the prevalence of sleep disorders and their impact on cognitive decline. A self-designed questionnaire of 10 items collected data regarding sleep quality, environmental influences, and daily stressful life events: participants filled out these questions regarding sleep, mood, stress, environmental factors, and lifestyle behaviors at certain times during the day. Questions were scored from 1 to 5 numerically regarding assessing sleep quality, yes/no for the presence or absence of disturbances and environmental factors causing stress, and open-ended descriptions of any possible disruptions. Participants answered the questions three times a day morning: upon waking, midday: around lunch, and night: before bedtime. To evaluate participants' cognitive functioning, we used the Mini-Mental State Examination (MMSE) (see Supplementary material 1 in the Appendices), which was developed by Folstein et al. in 1975 [21]. MMSE evaluated various cognitive functions, including orientation, registration, attention, calculation, recall, language, and visuospatial skills. The total score is 30 points, whereas the score below 24 indicates cognitive impairment. A structured questionnaire was administered (physically) to collect demographic information (age, gender, education, marital status, and residential area), and then MMSE was administered.

Informed consent was provided before data collection to ensure all participants understood the study aims, procedures, and their right to withdraw. Before starting this research, the Institutional Review Board (IRB) of Brain Tech Research Center approved it with IRB-2024-0026. Each participant was above 18, so parental or guardian consent was unnecessary. Confidentiality was maintained throughout the study. The data was used for research purposes only.

The analysis used IBM SPSS Statistics for Windows, Version 26 (Released 2019; IBM Corp., Armonk, New York, United States). The analytical and descriptive statistics were used with regression, chi-square, independent-sample t-test, mean, and standard deviation. The level of significance of p < 0.05 was used.

## Results

Table 1 represents an analysis examining the relationships between demographic variables, cognitive functioning, and sleep patterns. Gender distribution showed that males constituted 57 (38.0%) of the sample, with most participants exhibiting normal cognition (n = 37), while fewer experienced mild (n = 8) and moderate cognitive impairments (n = 12). Males' sleep patterns were distributed as very short sleep (n = 9), short sleep (n = 20), optimal sleep (n = 22), and long sleep (n = 6), with a significant association observed in sleep patterns (p = 0.03, χ² = 4.76). Females comprised 93 (62.0%) of the participants, primarily exhibiting normal cognition (n = 57), with smaller proportions experiencing mild (n = 6) and moderate impairments (n = 30). Sleep patterns for females showed no significant differences, with most falling into optimal and short sleep categories. Educational levels revealed that high school graduates accounted for 17 participants (11.3%) in the sample, with a significant association between education and cognitive impairment (p = 0.12, χ² = 13.3). Most high school graduates exhibited moderate cognitive impairment (n = 11), while fewer showed normal cognition (n = 6). This group's sleep patterns were predominantly short (n = 5) and short (n = 6). Bachelor's degree holders make up the majority (n = 114, 76.0%), with a large portion categorized as having normal cognition (n = 75), followed by mild (n = 12) and moderate impairments (n = 27). Their sleep patterns were concentrated in optimal sleep (n = 59). Master's degree holders (n = 19, 12.7%) followed a similar trend, with most participants categorized as normal cognition and distributed across all sleep categories. Age distribution indicated that middle-aged adults comprised 103 (68.7%) participants, with a significant association between age and cognitive functioning (p = 0.00, χ² = 135.0). Most participants in this group exhibited normal cognition (n = 94), with mild impairments (n = 9) and no moderate impairments. Middle-aged adults' sleep patterns were primarily optimal (n = 48), followed by short (n = 35). Older adults (n = 47, 31.3%) predominantly exhibited moderate cognitive impairment (n = 42), with no participants in the normal cognition category. Sleep patterns among older adults were mainly optimal sleep (n = 20) and short sleep (n = 10), with fewer in the long and very short sleep categories. Marital status analysis revealed that singles constituted 44 participants (29.3%) in the sample, with normal cognition (n = 35) as the most common category, followed by mild (n = 4) and moderate impairments (n = 5). Sleep patterns among singles were distributed as short sleep (n = 16) and optimal sleep (n = 21). Married participants, who accounted for 106 participants (70.7%) in the sample, were significantly associated with cognitive impairment (p = 0.01, χ² = 8.98), with most falling into the normal cognition (n = 59) and mild impairment (n = 10) categories. Their sleep patterns were primarily optimal (n = 47) and short (n = 29). These findings highlight significant associations between demographic variables, cognitive functioning, and sleep patterns. Middle-aged adults and individuals with higher education levels exhibited better cognitive functioning. Chi-square values underscored significant associations between age, education, and marital status with cognitive functioning, suggesting that these factors influence cognitive health and sleep behaviors.

**Table 1:** Descriptive statistics of demographic variables n: frequency; %: percentage; p: significance

Variables	n (%)	Normal cognition	Mild cognitive impairment	Moderate cognitive impairment	χ2	p-value	Very Short Sleep	Short Sleep	Optimal Sleep	Long sleep	χ2	p-value
Gender												
Male	57 (38.0%)	37	8	12	3.83	0.14	9	20	22	6	4.76	0.03
Female	93 (62.0%)	57	6	30			7	25	46	15		
Education												
High school	17 (11.3%)	6	0	11	13.3	0.12	6	5	4	2	22.7	0
Bachelor’s	114 (76.0%)	75	12	27			5	34	59	16		
Master’s	19 (12.7%)	13	2	4			5	6	5	3		
Age												
Middle Adults	103 (68.7%)	94	9	0	135	0	8	35	48	12	5.74	0.01
Old adults	47 (31.3%)	0	5	42			8	10	20	9		
Marital status												
Single	44 (29.3%)	35	4	5	8.98	0.01	4	16	21	3	3.35	0.34
Married	106 (70.7%)	59	10	37			12	29	47	18		

Table 2 shows the correlation analysis examining the relationships between age, sleep quality, mental state, and ecological factors. The results indicate a significant negative correlation between mental state and age (r = -0.90, p < 0.01), suggesting that as age increases, mental state outcomes tend to worsen. Sleep quality was not significantly correlated with age (r = -0.10), indicating no substantial relationship between these variables. A significant negative correlation was also observed between sleep quality and ecological factors (r = -0.79, p < 0.05), implying that poorer ecological conditions are associated with lower sleep quality. However, the relationship between mental state and ecological factors was weak and nonsignificant (r = -0.02). Similarly, no significant correlation existed between sleep quality and mental state (r = 0.10). These findings suggest that age and ecological factors play critical roles in influencing mental state and sleep quality, respectively, while the relationships between other variables remain less pronounced.

**Table 2:** Intercorrelations between study variable *p < 0.05, **p < 0.01 considered significant

Variables	Age	Sleep quality	Mental state	Ecological factors
Age	-	-	-	-
Sleep quality	^-0.10^	-	-	-
Mental state	-0.90^**^	0.10	-	-
Ecological factors	^-0.12^	-0.79^*^	-0.02	-

Table 3 highlights the differences in sleep quality, duration, mental state, and ecological factors across gender and marital status using independent sample t-tests. The gender comparison reveals minimal differences across variables. Males exhibited slightly better sleep quality and mental state scores than females, though these differences were not statistically significant. Females showed marginally longer sleep duration and slightly higher ecological factor scores, which also lacked statistical significance. Overall, the negligible effect sizes across gender groups indicate that gender does not significantly influence these variables within the study sample. The marital status comparison, however, yielded more pronounced findings. Single participants demonstrated significantly better mental state scores than married individuals, with a medium effect size indicating a meaningful difference. In contrast, sleep quality, duration, and ecological factors showed negligible differences between single and married participants, with no statistically significant relationships. These results suggest that marital status, explicitly being single, is associated with better mental state outcomes, while other variables remain unaffected by marital status. This pattern underscores the potential influence of social and relational contexts on mental health, with single individuals possibly benefiting from less stress or responsibilities tied to marital life. However, the small effect sizes for other variables suggest that broader demographic or contextual factors may mediate their relationships with gender and marital status.

**Table 3:** Comparison of gender and marital status across study variables using independent sample t-tests M: mean; SD: standard deviation; LL: lower limit; UL: upper limit; CI: confidence interval

Gender comparison									
Variables	Male		Female		t(148)	P	95% CI		Cohen’s d
	M	SD	M	SD	-	-	LL	UL	-
Sleep quality	3.23	1.07	3.13	1.22	0.51	0.61	-0.29	0.49	0.09
Sleep duration	6.56	2.56	7.02	1.87	-1.27	0.21	-1.18	0.26	-0.21
Mental state	24.02	6.24	22.14	7.45	1.59	0.11	-0.45	4.21	0.27
Ecological factor	21.19	2.03	21.42	2.09	-0.65	0.52	-0.91	0.46	-0.11
Comparison among marital status									
Variables	Single		Married		t(148)	p	95% CI		Cohen’s d
	M	SD	M	SD	-		LL	UL	-
Sleep quality	3.09	1.07	3.20	1.20	-0.51	0.61	-0.52	0.31	-0.09
Sleep duration	6.75	2.61	6.89	1.96	-0.35	0.73	-0.91	0.63	-0.06
Mental state	25.43	5.47	21.78	7.37	2.96	0.004	1.21	6.09	0.53
Ecological factor	21.41	2.14	21.30	2.04	0.29	0.77	-0.63	0.84	0.05

Table 4 compares education levels (high school, bachelor's, and master's) across ecological factors, sleep quality, and mental state using one-way ANOVA. The analysis revealed no significant differences in ecological factors and sleep quality across education levels, as indicated by small effect sizes (η² = 0.007 and η² = 0.016, respectively) and nonsignificant F-values. Participants across all education levels demonstrated similar scores for ecological factors, with means ranging from 21.24 to 21.65. Similarly, sleep quality scores were comparable, with slightly higher mean scores observed for high school graduates (M = 3.24 ± 1.48) compared to bachelor's (M = 3.22 ± 1.09) and master's degree holders (M = 2.79 ± 1.27). In contrast, a significant difference was observed in mental state scores across education levels (F(2,147) = 3.63, p < 0.05, η² = 0.047). Bachelor's and master's degree holders had notably higher mental state scores (M = 23.39 ± 6.80 and M = 23.47 ± 6.57, respectively) than high school graduates (M = 18.59 ± 8.15). The medium effect size suggests that education level plays a meaningful role in shaping mental state outcomes, with higher education being associated with better mental state scores. Overall, the results indicate that while education level does not significantly influence ecological factors or sleep quality, it is significantly associated with mental state, highlighting the potential cognitive benefits of higher education.

**Table 4:** Comparison of education level across different variables SD: standard deviation, η²: effect size *p < 0.05 considered significant (calculated by ANOVA)

Variables	High school	Bachelor’s	Master’s	F(2,147)	η²
	Mean (±SD)	Mean (±SD)	Mean (±SD)	-	-
Ecological factors	21.65 ± 2.18	21.24 ± 2.03	21.63 ± 2.22	0.52	0.007
Sleep quality	3.24 ± 1.48	3.22 ± 1.09	2.79 ± 1.27	1.15	0.016
Mental state	18.59 ± 8.15	23.39 ± 6.80	23.47 ± 6.57	3.63*	0.047

**Figure 1:**
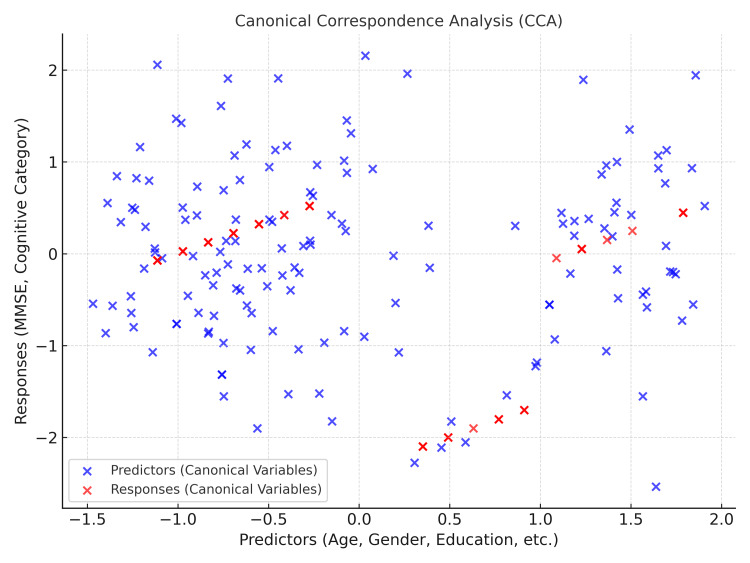
Canonical correspondence analysis (CCA) plot MMSE: Mini-Mental State Examination

Figure 1 displays the canonical correspondence analysis (CCA) plot, highlighting the relationships between predictors (age, gender, education, marital status, sleep quality, sleep duration, and ecological factors) and response variables (mental state and cognitive category). The blue points represent the predictors, while the red points denote the response variables. The distribution of points across the two canonical components demonstrates the strength and direction of associations between the variables. Predictors such as age, sleep quality, and ecological factors contribute to variations in mental state and cognitive category. The proximity of red and blue points suggests a stronger relationship, whereas greater distances imply weaker associations. This visualization helps understand how demographic and health-related predictors influence mental state outcomes and cognitive categories, concisely representing multivariate relationships within the dataset. The CCA plot supports identifying significant predictors that impact mental state and cognitive performance, offering valuable insights for further analysis and interpretation.

## Discussion

The findings of this study highlight significant associations between ecological factors, sleep disorders, and cognitive decline. Our results confirm prior research emphasizing the detrimental impact of poor sleep on cognitive functioning, reinforcing that sleep disturbances exacerbate cognitive decline, particularly in older adults [1,2]. The study underscores that sleep duration, quality, and environmental conditions play critical roles in cognitive health. These results align with prior findings that sleep durations below 4 or above 10 hours accelerate cognitive deterioration [16]. Additionally, actigraphy measures demonstrated that waking after sleep onset is strongly associated with worsening cognitive performance over time [17,18].

Ecological factors such as environmental noise, air pollution, and temperature significantly impacted sleep quality in our study. Research has previously established that chronic exposure to environmental noise, particularly transportation and aircraft noise, disrupts sleep architecture and increases the risk of long-term cognitive impairment [21,22], aligning with prior research indicating that air pollution is a risk factor for neurodegenerative diseases and dementia [15,23].

A study found age and educational background to be strong predictors of cognitive function. Middle-aged adults exhibited better cognitive outcomes than older participants, corroborating earlier research showing age-related cognitive decline as a function of disrupted sleep patterns [24,25]. Furthermore, individuals with higher education levels demonstrated superior cognitive performance, supporting the cognitive reserve hypothesis, which posits that education is a protective factor against cognitive deterioration [26].

This study's findings suggest that psychosocial factors, including marital status, contribute to sleep disturbances and cognitive outcomes. Single individuals in our study exhibited better cognitive function than married participants, consistent with research indicating that marital stress and caregiving responsibilities can contribute to sleep disruption and cognitive decline [11].

This study has several limitations. The cross-sectional design restricts the ability to establish causality between ecological factors, sleep disorders, and cognitive decline, necessitating longitudinal studies to confirm these associations over time. Reliance on self-reported sleep quality and environmental conditions introduces the possibility of recall bias and subjective variability, highlighting the need for objective measures such as polysomnography and environmental monitoring to enhance validity. Additionally, while the sample size is adequate, it may not fully capture the diversity of ecological influences across different socioeconomic and geographic contexts, emphasizing the importance of larger, more diverse populations to improve generalizability. Potential confounding variables, including pre-existing medical conditions, genetic predispositions, and lifestyle habits, were not exhaustively controlled, which may have influenced the observed associations. Addressing these factors in future research will provide a more comprehensive understanding of the interplay between sleep, environment, and cognitive function. Given the observed associations, targeted interventions to improve sleep quality through environmental modifications are necessary, including policies to reduce noise pollution, improve air quality, and optimize indoor temperature regulation. Future research should also explore longitudinal assessments to more definitively establish causal relationships between sleep disorders and cognitive impairment.

## Conclusions

This study underscores the significant role of ecological and environmental factors in shaping sleep health and cognitive function. Findings reveal that poor sleep quality, influenced by noise pollution, air quality, and temperature fluctuations, contributes to cognitive decline, particularly in older adults. The results highlight the bidirectional relationship between sleep disturbances and cognitive impairment, reinforcing the need for targeted interventions that address modifiable environmental stressors. The findings indicate that cognitive impairment is significantly associated with age, education level, and marital status. Older adults are more likely to experience moderate cognitive impairment, while those with lower education levels show higher rates of cognitive decline. Additionally, married individuals tend to have better cognitive outcomes compared to those who are single.

Addressing sleep-related ecological challenges through public health policies, urban planning, and environmental modifications could mitigate the risk of sleep disorders and their long-term cognitive consequences. Future research should explore longitudinal relationships between ecological influences and cognitive decline, incorporating objective sleep measures and diverse populations to strengthen these findings. Society can develop more effective strategies to reduce cognitive impairment and improve overall well-being by prioritizing sleep as a critical component of cognitive health.

## Appendices

 Supplementary material 1

**Table 5:** Mini-Mental State Examination (MMSE) Credit: This has been adapted from the Mini-Mental State Examination (MMSE) as originally described by Folstein et al. (1975). Reference: [21]

Section	Task	Points
Orientation	What is the (year) (season) (date) (day) (month)?	5
-	Where are we (state) (country) (town) (hospital) (floor)?	5
Registration	Name 3 objects: 1 second to say each. Then ask the patient all 3 after you have said them. Give 1 point for each correct answer. Then repeat them until he/she learns all 3. Count trials and record. Trials	3
Attention and Calculation	Serial 7's. 1 point for each correct answer. Stop after 5 answers. Alternatively, spell "world" backward	5
Recall	Ask for the 3 objects repeated above. Give 1 point for each correct answer	3
Language	Name a pencil and watch	2
-	Repeat the following “No ifs and buts"	1
-	Follow a 3-stage command: “Take a paper in your hand fold it in half and put it on the floor”	3
-	Read and obey the following: CLOSE YOUR EYES	1
-	Write a sentence	1
-	Copy the design shown	1
Total	-	30
